# Expression of CD40 and CD40L in Gastric Cancer Tissue and Its Clinical Significance

**DOI:** 10.3390/ijms10093900

**Published:** 2009-09-04

**Authors:** Rui Li, Wei-Chang Chen, Xue-Qin Pang, Chen Hua, Ling Li, Xue-Guang Zhang

**Affiliations:** 1 Department of Gastroenterology, First Affiliated Hospital of Soochow University; Key Laboratory of Medicine and Clinical Immunology of Jiangsu Province, Suzhou 215006, China; E-Mail:liruidoc@gmail.com (R.L.); 2 Department of Emergency, Second Affiliated Hospital of Soochow University, Suzhou 215004, China; 3 Institute of Medical Biotechnology, Soochow University, Suzhou 215007, China

**Keywords:** gastric cancer, CD40, CD40L, apoptosis, CD138

## Abstract

To study expression of CD40 and CD40L in gastric cancer tissue we assessed gastric cancer patients admitted to the Department of Gastroenterology of The First Affiliated Hospital of Soochow University and control subjects. Gastric cancer and normal (from around tumours) tissue samples were obtained from patients. Venous blood samples (gastric cancer and ulcer groups) were drawn on the morning of the day before surgery for the measurement of peripheral sCD40L. The expression of CD40 in gastric carcinoma specimens was examined immuno-histochemically. The clinicopathological factors, including age, sex, tumor size, gross appearance, degree of cellular differentiation, histological classification, depth of tumor invasion, lymph node metastasis, peritoneal dissemination, and TNM stage were analyzed according to the different expression of CD40. The results indicated a high CD40 expression in gastric cancer tissues. This positive expression of CD40 revealed a significant (P < 0.05) correlation with lymphatic metastasis and tumor TNM stage in gastric cancer patients. It is concluded that higher CD40 expression existed in expanding type tumors and could play an important role in clinical diagnosis of gastric cancer patients.

## 1. Introduction

Patients with advanced gastric carcinoma, especially serosa-invading tumours, have a poor prognosis, even after curative resection. In these cases, peritoneal dissemination originating from free cancer cells seeded from primary gastric cancer often occurs after surgery and is the most common type of recurrence [1]. To date, various treatments have been used for peritoneal dissemination of gastric cancer, but there is no effective therapy for this condition.

With the advances in molecular biology, much progress has been made in our understanding of the molecular pathogenesis of cancer. Cell surface protein CD40, whch is mainly expressed in B-cells and other antigen-presenting cells, plays a critical role in B-cell activation by providing cell survival signals via interaction with the CD40 ligand (CD40L) expressed on the surface of activated T-cells [2]. CD40 ligand (CD40L) is a transmembrane protein of the tumor necrosis factor (TNF) family, originally identified in T cells. CD40L and its receptor, CD40, have been implicated in the pathogenesis of atherosclerosis. In addition, occurrence and development of gastric cancer is still closely related to the CD138, gelsolin, P53 and NF-κB molecules. Syndecan-1 (CD138) is the most well-characterized member of the family and its expression is localized entirely in epithelial cells, with stratified squamous epithelia showing the most abundant expression. Decreased expression of syndecan-1 has been previously reported to correlate with increased tumorigenicity and with tumor invasion and progression [3]. Syndecan-1 binds to various ECM components, such as collagen, fibronectin, thrombospondin, and tenascin, via its HS-GAG, and most of its biological functions are considered to be associated with this process. Gelsolin is down-regulated in many types of solid tumors, including breast, lung, gastric, bladder, colorectal, and prostate cancers, and is associated with disease progression. Conversely, overexpression of gelsolin has been found to be a negative prognostic predictor in a subpopulation of patients with non-small cell lung cancer, urothelial cancer, and EGFR +/erbB-2 + breast cancers [4]. P53 has been associated with 60% of all cancers, and in gastric cancer, mutations and/or overexpression of the mutant p53 protein has been found in up to 76% of tumor samples [5]. Abnormalities of p53 have also been found in dysplastic tissue and intestinal metaplasia (a suspected precursor of gastric cancer).

Tumor cell metastasis and invasion is the main reason for death of gastric cancer patients. The objective of the study was to examine how CD40-CD40L expression affects the biological behavior of gastric cancer. We first examined the expression and functions of CD40 and CD40L in gastric carcinoma tissues and analysed the correlation of CD40-CD40L with tumor cell metastasis and invasion. Then we investigated CD138, gelsolin, P53 and NF-κB expression in gastric cancer tissues and their correlation with CD40. As a result, molecular therapeutic interventions directed at gastric cancer might be clinically effective in preventing metastasis in gastric cancer.

## 2. Methods

### 2.1. Human Gastric Cancer Specimens

Between September 2002 and March 2003, 56 patients at the Department of Gastroenterology (The First Affiliated Hospital of Soochow University) were assessed. Informed consent was obtained preoperatively from each patient to use part of the resected cancer lesions for research. The patient group comprised 37 men and 19 women, with a mean age of 57 years (range: 36 to 76 years). None of the gastric cancer patients had received preoperative neoadjuvant chemotherapy or radiotherapy.

According to the TNM classification of the International Union Against Cancer (UICC), there were seven stage I, 12 stage II, 28 stage III, and nine stage IV gastric cancers and one grade 1, 19 grade 2, 26 grade 3, and six grade 4 tumors. Gastric cancer tissue samples were obtained from all 56 patients. Normal tissue samples were collected from around tumour tissues. Immediately upon surgical resection, tissue samples were fixed in 5% formaldehyde solution for 12 to 24 hours and paraffin embedded for subsequent analysis. All studies were approved by the Human Subjects Committee of Soochow University.

### 2.2. Human Serum Specimens

Between September 2002 and March 2003, 45 gastric cancer patients at the Department of Gastroenterology, the First Affiliated Hospital of Soochow University were selected. The group comprised 32 men and 13 women with a mean age of 55 years (range: 38 to 76 years). None of the gastric cancer patients had received preoperative neoadjuvant chemotherapy or radiotherapy. According to the TNM classification of the International Union Against Cancer (UICC), there were six stage I, 11 stage II, 22 stage III, and six stage IV gastric cancers. The gastric ulcer group comprised seven men and three women with a mean age of 42 years (range: 24 to 61 years). The healthy group (control group) comprised 11 men and four women with a mean age of 48 years (range: 21 to 59 years). There were no significant differences between these groups with regards to mean age and sex proportion.

Venous blood samples (gastric cancer and ulcer groups) for the measurement of peripheral sCD40L were drawn on the morning of the day before surgery. Venous blood samples for the measurement of peripheral sCD40L were collected from each volunteer (healthy group) and all volunteers were asked to come for sample collection after at least 12 h of fasting. These blood samples were centrifuged at 5,000 rpm for 5 min during the operative day, the serum and plasma samples were stored at −70 °C until they were used for measurement of sCD40L.

### 2.3. Immunohistochemistry

Immunohistochemical analysis was performed on formalin-fixed, paraffin-embedded pancreatic specimens, including one autoimmune pancreatitis and one well-differentiated ductal adenocarcinoma. 5-μm sections were cut onto coated slides and were deparaffined using routine techniques.

After treatment with 1% hydrogen peroxidase for 10 min to block endogenous peroxidases, the sections were subsequently incubated with monoclonal antibodies (anti-CD40 (1:40), anti-CD138 (1:50), Gelsolin antibody (1:50), mouse anti-Human P53 monoclonal antibody, mouse anti-Human NF-κB/P65 monoclonal antibody) for 2 h at room temperature. Labelling was detected by streptavidin-biotin-peroxidase complex (Dako, Merelbeke, Belgium). Negative control was obtained by omitting the primary antibody.

Sample mixed with only PBS buffer was treated as negative control. Hodgkin's lymphoma served as positive controls for mouse anti-Human CD40 monoclonal antibody. Specimens offered by the reagent company were used as positive controls for mouse anti-Human Gelsolin, P53, and P65 monoclonal antibodies, respectively.

### 2.4. Measurement of Apoptotic Cells

The proportion of apoptotic tumor cells was examined by the TUNEL (Terminal deoxynucleotidyl transferase (TdT)-mediated deoxyuridine triphosphate (dUTP)-digoxigenin nick end labelling) assay [6] using *In Situ* Cell Death Detection Kit (Roche Applied Science, Penzberg, Germany). All measurements were performed according to the manufacturer’s instructions. Briefly, air-dried cell samples were fixed with a freshly prepared Fixation Solution for 1 h at 15 to 25 °C. Then tumor cell nuclei were stripped from proteins by incubation with 20 mg/mL proteinase K for 15 min at RT. Endogenous peroxidase was inactivated by covering the sections with 2% H_2_O_2_ for 10 min at RT. The sections were digested with 20 μg/mL proteinase K for 15 min at 37 °C, presoaked in labeling buffer for 10 min, and then incubated with TDT and DIG-d-UTP solution at 37 °C for 60 min covered with parafilm in a humidified chamber. The sections were rinsed with distilled water, covered with 2% aqueous solution of bovine serum albumin for 10 min at RT. The sections were then covered with extra-avidin peroxidase at 37 °C for 30 min. Slides were counter stained with hematoxylin. The cells were defined as apoptotic if the nuclear area of cells was labeled positively (red color). To quantify the apoptotic events, the number of cells undergoing apoptosis was counted in five randomly selected fields in each slide under 20 × 10 magnification. The number of positively labeled nuclei per total nuclei in those fields was expressed as the apoptotic index (AI). AI = (the number of apoptotic cells/total number of cells) × 100%.

### 2.5. Immunostaining Score

Semi-quantitative count of the cells staining was scored according to Barnes’ method. Assessment of score standard should be based on three parameters:

- staining intensity,
- number of positive cells,
- score1 (staining intensity) × score2 (number of positive cells).

The intensity of immunostaining was assessed by 2 different observers, with respect to the staining intensity into the following 4 grades: −, no staining (negative); +, faintly stained up to 25% (weak positive); ++, 25% to 50% cells moderately stained (moderate positive); +++, 50% or more cells markedly stained (strong positive).

### 2.6. Enzyme Immunoassays

Specific immunoassays for sCD40L (sCD40 detection limit, 95 pg/mL; with intra- and interassay CVs < 10% at different levels of sCD40L, Bender Medsystems) was measured using a commercial immunoassay kit (Bender MedSystems, Vienna, Austria).

### 2.7. Statistical Analysis

Differences in the results between groups were analyzed by the Mann–Whitney U-test, with a p-value of less than 0.05 being taken as significant. Correlations were analyzed with Spearman's rank correlation coefficient test.

## 3. Results

### 3.1. CD40, CD138, Gelsolin, P53, P65 Expression in Gastric Cancer Tissue and Normal Tissue

**Figure 1.**
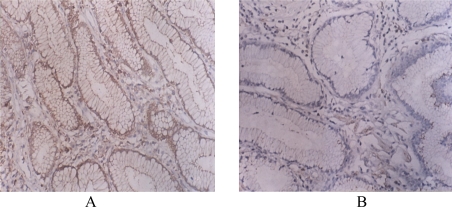

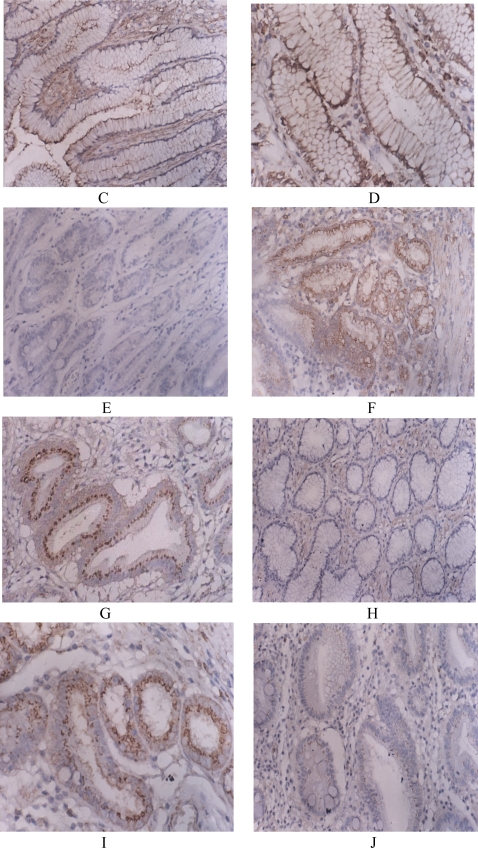
A. Strong positive expression of CD40 in gastric cancer tissues; B. Negative expression of CD40 in normal tissues around tumor. C. Weak positive expression of CD138 in gastric cancer tissues; D. Positive expression of CD138 in normal tissues around tumor; E. Negative expression of Gelsolin in gastric cancer tissues; F. Strong positive expression of Gelsolin in normal tissues around tumor; G. Positive expression of P53 in gastric cancer tissues; H. Negative expression of P53 in normal tissues around tumor; I. Positive expression of P65 in gastric cancer tissues; J. Negative expression of P65 in normal tissues around tumor.

In the gastric cancer group (56 patients), 19 patient (33.9%) had positive expression of CD40 and 10 patients (17.9%) had strong positive expression of CD40. Negative expression of CD40 was observed in normal tissues (from around the tumours). The expression rate of CD40 in gastric cancer tissues was significantly stronger (P < 0.01) than that in normal tissues (Table 1 and Figures 1A, B); Positive expression of CD138 in tumour and normal tissues was observed in 44.6% and 96.9% of cases, respectively (Table 1 and Figures 1C, D); Positive expression of Gelsolin in tumour and normal tissues was observed in 10.7% and 81.3% of cases, respectively (Table 1 and Figures 1E, F); Positive expression of P53 and P65 in tumour tissues was observed in 69.6% and 64.3% of cases, respectively (Table 1 and Figures 1G, H, I, J).

### 3.2. Correlation of CD40, CD138, Gelsolin, P53 and P65 Expression with Clinical Pathological Factors in Patients with Gastric Cancer

**Figure 2.**
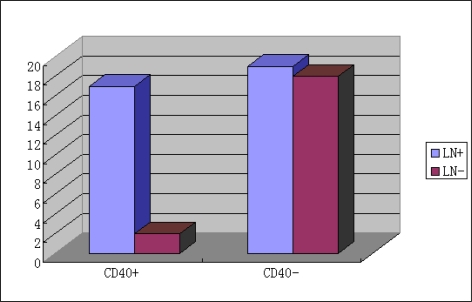
Correlation of CD40 expression with lymphatic metastasis.

**Figure 3.**
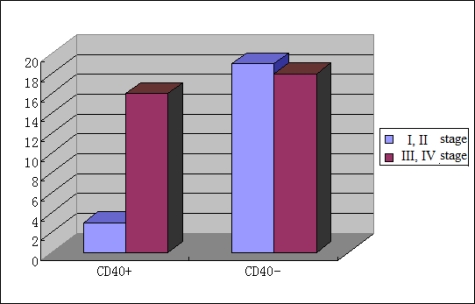
Correlation of CD40 expression with tumor TNM stage.

As shown in Figures 2 and 3, positive expression of CD40 revealed no correlation (P > 0.05) with differentiation, size and invasion of tumors, but showed a significant (P < 0.05) correlation with lymphatic metastasis and tumor TNM stage in gastric cancer patients. Table 2 shows univariate analysis of the relationship of CD40 to clinical and pathological factors. In relation to the lymphatic and tumour metastasis and TNM stage, a significant differences of positive expression of CD40 were seen between patients with and without lymphatic metastasis (p < 0.01), between patients with and without tumour metastasis (p < 0.01), and between stage I, II and stage III, IV patients (p < 0.05). No significant (P > 0.05) correlation was demonstrated between positive expression of CD40 and clinical progression, stage. As shown in Table 3, positive expression of CD138 and Gelsolin showed a significant (P < 0.05) correlation with lymphatic metastasis in gastric cancer patients. However, no significant (P > 0.05) correlation was demonstrated between positive expression of CD138 and Gelsolin and tumors size. Positive expression of CD138 revealed a significant correlation (P < 0.05) with tumor differentiation and tumor TNM stage in the gastric cancer patients. Positive expression of P53 showed a significant (P < 0.05) correlation with tumor differentiation, but showed no significant (P > 0.05) correlation with lymphatic metastasis and tumor invasion. As shown in Table 3, positive expression of P65 showed a significant (P < 0.05) correlation with tumor differentiation, size and invasion and lymphatic metastasis in the gastric cancer patients.

### 3.3. Correlation of CD40 Expression with CD138, Gelsolin, P53 and P65 Expression in Gastric Cancer Tissues

As shown in Table 4, a significant negative correlation was found between CD40 and CD138 expression (r = 0.375, P < 0.05). Likewise, a highly significant positive correlations existed between expression of CD40 and expression of P53 and P65 (r = 0.201, P < 0.01, r = 0.277, P < 0.01, respectively). Spearman's rank correlation coefficient test still showed that there is no correlation between CD40 and gelsolin expression.

### 3.4. Correlation of CD40 Expression with Apoptotic Index (AI) in Gastric Cancer Tissues

**Figure 4.**
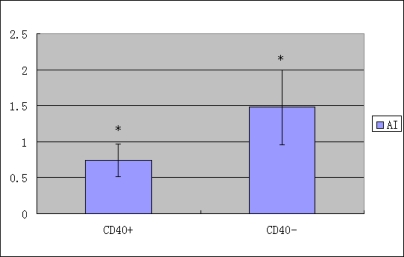
Correlation of CD40 expression with apoptotic index (AI) in gastric cancer tissues, *P < 0.05.

As shown in Table 5 and Figure 4, apoptotic index (AI) of the tumor cells was 1.16 ± 0.37. A significant difference (P < 0.50) was found between the AI (0.74 ± 0.23) of CD40 positive expression group and that (1.48 ± 0.52) of CD40 negative expression group. A significantly negative correlation was found between the CD40 expression and apoptotic index in the gastric cancer tissues (r = 0.263, P < 0.05).

### 3.5. sCD40L Level in Peripheral Blood

**Figure 5.**
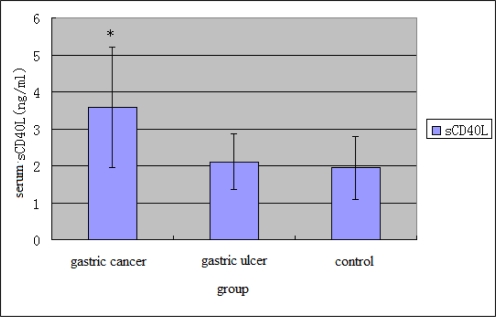
sCD40L levels in three different groups, values are mean ±S.D. *P < 0.05.

**Figure 6.**
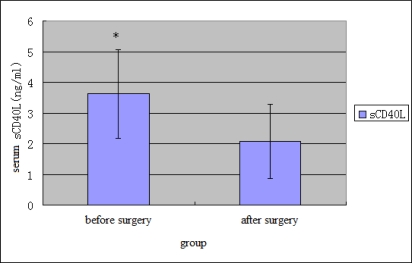
sCD40L levels in before and after surgery, values are mean ± S.D. *P < 0.05.

The results of the univariate analysis were summarized in Table 6. In relation to the invasion depth and TNM stage of the tumor, a significant difference of sCD40L level was seen between groups with different invasion depths (mucous membrane, muscular layer and serous membrane from inner to outer) (p < 0.05) and between stage I, II and stage III, IV patients (p < 0.05). In relation to the lymphatic metastasis and tumour metastasis, a significant difference of sCD40L level was seen between patients with lymphatic metastasis and patients without lymphatic metastasis (p < 0.01) and between patients with distant metastasis and patients without distant metastasis (p < 0.01). As shown in Table 6 and Figure 5, the level of sCD40L (3.57 ± 1.63ng/mL) in gastric cancer group was significantly (P < 0.01) higher than that of healthy group (1.94 ± 0.86ng/mL). Serum sCD40L level of gastric cancer patients after surgery was significantly (P < 0.01) decreased (Table 6 and Figure 6). In patients with gastric cancer, correlation was observed between serum sCD40L and lymphatic metastasis and tumors invasion and TNM stage.

## 4. Discussion

The importance of CD40–CD40L interactions has been well demonstrated in diseases such as X-linked hyper IgM syndrome, atherosclerosis, Hodgkin’s disease, and Alzheimer’s disease [7] and [8]. Previous studies have also reported that CD40L levels are elevated in colon cancer patients [9]. However, no data were available about CD40L levels in gastric cancer tissues. Recent studies have suggested that CD40 ligation can enhance cytotoxic T lymphocytes activity and reduce tumor growth. A member of the TNF-receptor superfamily, CD40 plays a central role in immune activation. To examine expression of CD40 in gastric cancer tissues, we tested the correlation of CD40 with lymphatic metastasis and tumors invasion. Our work revealed significantly increased CD40 expression in gastric cancer. We still found that immunohistochemical expression of CD40 was significantly associated with gastric cancer cells metastasis and invasion. These results indicate that CD40 expression can be considered to be a tumor progression-related factor and a prognostic factor in gastric carcinoma.

The corresponding ligand is CD40L. The ligand it expressed on activated T cells is one of the major participants in the interaction between T and B cells, and T-cell-antigenpresenting cells. The absence of the CD40–CD40L signal can be associated with severe immune deficiency, as has been shown in X-linked IgM hyperimmunoglobulinemia, in which there is absence of CD40L [10]. In addition to being a transmembrane protein, CD40L may be also detected as a soluble protein in serum [11]. These dynamic interactions between the CD40 and its ligand have raised the possibility of a therapeutic application. The blockade of the interaction between CD40 and CD40L due to mutation leads to severe defects in the humoral and cellular immunity, as was shown on the mice models. There can be potential benefit in the blockade of the interaction between CD40 and CD40L in the therapy of autoimmune diseases or the suppression of the allograft rejection [12]. The levels of sCD40L were higher among gastric cancer patients in comparison with healthy controls because it was also demonstrated in one previous study [13,14]. In addition, significant correlations between sCD40L level and tumor metastasis, invasion were found. This indicated that the high serum levels of this parameter are associated with the progression of tumor.

Wild-type p53 exerts its cell cycle control and apoptotic effects by acting as a transcription factor, upregulating expression of several genes including p21. In B-cell malignancies, where p53 mutations have been reported at 10 to 40%, abnormalities in p53 are associated with poor prognosis and reduced response to therapy [15,16]. A correlation between p53 protein expression and clinical effect could be evaluated in this study. The p53 expression level was determined by immunohistochemical staining of tissue samples from the primary tumour, which might not reflect the expression level in metastatic lesions. Furthermore, it was recently demonstrated that p53 protein expression are closely associated with CD40 expression in the tumour cells. CD40 and p53 genes would produce a synergistic augmentation of gastric cancer cell immunogenicity.

Syndecan-1/CD138 is expressed during murine B-cell development in the pre-B-cell stage, is lost in mature B cells and is re-expressed strongly in mature plasma cells [17]. A number of studies have investigated the expression of SDC-1 in human carcinomas. Decreasing SDC-1 expression was correlated with advanced tumour stage and an increased metastatic tendency of the cancer cells in a wide range of tumours. In contrast, increased SDC-1 expression and correlation with a higher degree of malignancy, as well as expression in connective tissue surrounding the tumour, has also been reported in other tumours. Our work showed that there is negative correlation between CD40 expression and CD138 expression in gastric cancer. This indicates that CD40 is a negative regulator of CD138 availability. Loss of epithelial syndecan-1 is a general feature of carcinoma progression. In agreement with other analyses of gastric carcinoma, loss of epithelial syndecan-1 correlated with tumor TNM stage and incidence of metastases to local lymph nodes [18]. Thus the loss of epithelial syndecan-1 is likely permissive for development of higher stage, more biologically aggressive tumors.

In this work, decreased gelsolin expression in gastric cancer tissue was correlated to the lymph node metastasis and tumours invasion. Therefore, decreased expression of gelsolin may contribute to the cell apoptosis. In addition, there isn’t a direct correlation between expression of CD40 and gelsolin. This indicated that gelsolin loss wasn’t regulated by CD40 expression.

CD40 can generate both proapoptotic and antiapoptotic signals. The present study demonstrated that there was significant negative correlation between CD40 expression and apoptotic index in gastric cancer tissue. This indicated that CD40 molecule affected gastric cancer occurrence and development by regulating apoptotic index. Further studies need to elucidate and confirm more precisely the roles and mechanisms for CD40L during the subsequent stages of gastric cancer development.

## 5. Conclusions

The results presented in this paper, together with our previous work, suggest that co-expression of CD40 and CD40L and resultant constitutive activation of the CD40 signal pathway are of primary importance in understanding the process of tumor invasion. This CD40 signaling suppresses apoptosis and promotes proliferation of infected cells. These findings imply that the potential therapeutics targeting CD40 signal pathway can be used to control the proliferation of tumor cells of gastric cancer. These findings may assist improved biomarker identification of aggressive forms of gastric carcinoma.

## Figures and Tables

**Table 1.** Expression of CD40, CD138, Gelsolin, P53 and P65 in gastric cancer and normal tissues.

**Group**		**Gastric Cancer Tissues**	**Normal Tissues**

**Total Cases**		56	32
**CD40 expression rate (case)**	−	37	30
	+	6	2
	++	3	0
	+++	10	0
	* P value		<0.01

**CD138 expression rate (case)**	−	31	1
	+	17	8
	++	6	9
	+++	2	14
	* P value		<0.01

**Gelsolin expression rate (case)**	−	50	6
	+	5	13
	++	1	5
	+++	0	8
	* P value		<0.05

**P53 expression rate (case)**	−	17	28
	+	11	3
	++	16	1
	+++	12	0
	* P value		<0.01

**P65 expression rate (case)**	−	20	25
	+	21	4
	++	7	3
	+++	8	0
	* P value		<0.05

* P < 0.01, compared with gastric cancer (tissues) group.

**Table 2.** Univariate analysis of relationship of CD40 expression with clinical pathological factors in patients with gastric cancer.

**Clinical pathological factors**	**Cases (n)**	**CD40 positive expression rate (%)**	**P value**
Sex			>0.05
Man	37	29.7 (11)	
Woman	19	42.1 (8)	
Age (years)			>0.05
≥50	43	30.2 (13)	
<50	13	46.2 (6)	
Tumor position			>0.05
Cardia	12	16.7 (2)	
Corpora ventriculi	24	41.7 (10)	
Sinuses ventriculi	20	35.1 (7)	
Tumor size (cm)			>0.05
≥5	31	32.3 (10)	
<5	25	36.3 (9)	
Tissue differentiation			>0.05
High differentiation	8	50 (4)	
Middle differentiation	37	24.3 (9)	
Low differentiation	11	54.5 (6)	
Invasion depth			>0.05
Mucous membrane	5	40 (2)	
Muscular layer	28	32.1 (9)	
Serous membrane from inner to outer	23	34.8 (8)	
Blood-vessel invasion			>0.05
Yes	27	48.1 (13)	
No	29	20.7 (6)	
Lymphatic metastasis			<0.01
Yes	36	47.2 (17)	
No	20	10 (2)	
Distant metastasis			<0.01
Yes	9	100 (9)	
No	47	(21.3) 10	
TNM stage			<0.05
I, II	22	13.6 (3)	
III, IV	34	47.1 (16)	

**Table 3.** Univariate analysis of relationship of CD138, Gelsolin, P53 and P65 expression with clinical pathological factors in patients with gastric cancer.

**Clinical pathological factors**	**Cases(n)**	**CD138 positive expression Rate (%)**	**P value**	**Gel positive expression Rate (%)**	**P value**	**P53 positive expression Rate (%)**	**P value**	**P65 positive expression Rate (%)**	**P value**
**Sex**			>0.05		>0.05		>0.05		>0.05
**Man**	37	43.2 (16)		2.7 (1)		64.9 (24)		59.5 (22)	
**Woman**	19	47.4 (9)		26.3 (5)		78.9 (15)		73.7 (14)	
**Age (years)**			>0.05		>0.05		>0.05		>0.05
**≥50**	43	48.8 (21)		11.6 (5)		72.1 (31)		62.8 (27)	
**<50**	13	30.8 (4)		7.7 (1)		61.5 (8)		69.2 (9)	
**Tumor position**			>0.05		>0.05		>0.05		>0.05
**Cardia**	12	58.3 (7)		8.3 (1)		16.7 (2)		58.3 (7)	
**Corpora ventriculi**	24	33.3 (8)		8.3 (2)		79.2 (19)		41.7 (10)	
**Sinuses ventriculi**	20	50 (10)		15 (3)		90 (18)		95 (19)	
**Tumor size (cm)**			>0.05		>0.05		<0.05		<0.05
**≥5**	31	48.4 (15)		6.5 (2)		93.5 (29)		90.3 (28)	
**<5**	25	40 (10)		16 (4)		40 (10		32 (8)	
**Tissue**			<0.05		>0.05		<0.01		<0.05
**High**	8	87.5 (7)		37.5 (3)		25 (2)		25 (2)	
**Middle**	37	45.9 (17)		5.4 (2)		73 (27)		62.2 (23)	
**Low differentiation**	11	9.1 (1)		9.1 (1)		90.9 (10)		100 (11)	
**Invasion depth**			<0.05		<0.05		>0.05		<0.01
**Mucous membrane**	5	100 (5)		80 (4)		20 (1)		20 (1)	
**Muscular layer**	28	64.3 (18)		3.6 (1)		67.9 (19)		53.6 (15)	
**Serous membrane from inner to outer**	23	8.7 (2)		4.3 (1)		82.6 (19)		87 (20)	
**Blood-vessel invasion**			>0.05		>0.05		>0.05		<0.05
**Yes**	27	37 (10)		7.4 (2)		70.4 (19)		92.6 (25)	
**No**	29	51.7 (15)		13.8 (4)		69 (20)		37.9 (11)	
**Lymphatic metastasis**			<0.01		<0.01		>0.05		<0.01
**Yes**	36	19.4 (7)		2.8 (1)		72.2 (26)		88.9 (32)	
**No**	20	90 (18)		25 (5)		65 (13)		20 (4)	
**Distant metastasis**			<0.01		>0.05		>0.05		>0.01
**Yes**	9	0 (0)		0 (0)		100 (9)		100 (9)	
**No**	47	53.2 (25)		12.8 (6)		63.8 (30)		57.4 (27)	
**TNM stage**			<0.05		>0.05		>0.05		<0.01
**I, II**	22	86.4 (19)		22.7 (5)	>0.05	63.6 (14)		27.3 (6)	
**III, IV**	34	17.6 (6)		2.9 (1)		73.5 (25)		88.2 (30)	

**Table 4.** Correlation of CD40 with relative molecules in gastric cancer tissues.

**Group**	**CD138**		**Gelsolin**		**P53**		**P65**	

	+	−	+	−	+	−	+	−
CD40 positive expression (n = 19)	4*	15	2	17	18**	1	19**	0
CD40 negative expression (n = 37)	21	16	4	33	21	16	17	20

Note:

* P < 0.05,

** P < 0.01.

**Table 5.** Correlation of CD40 expression with apoptotic index (AI) in gastric cancer tissues.

**Group**	**Cases (n)**	**AI (%)^a^**	**P value**
gastric cancer tissues	56	1.16 ± 0.37	
CD40 positive cancer tissues	19	0.74 ± 0.23	
CD40 negative cancer tissues	37	1.48 ± 0.52	<0.01

Note: P < 0.01, CD40 positive cancer tissues vs CD40 negative cancer tissues,

^a^ Values are the means ± S.D.

**Table 6.** Univariate analysis of relationship of serum sCD40L level with clinical pathological factors in patients with gastric cancer,

**Clinical pathological factors**	**Cases (n)**	**sCD40L (ng/ml)^a^**	**P value**
Sex			>0.05
Man	32	3.48 ± 1.74	
Woman	13	3.65 ± 1.52	
Age (years)			>0.05
≥50	36	3.39 ± 1.68	
<50	9	3.73 ± 1.45	
Tumor position			>0.05
Cardia	9	3.73 ± 1.45	
Corpora ventriculi	20	3.56 ± 1.64	
Sinuses ventriculi	16	3.44 ± 1.76	
Tumor size (cm)			>0.05
≥5	26	3.43 ± 1.80	
<5	19	3.62 ± 1.47	
Tissue differentiation			>0.05
High differentiation	6	3.35 ± 1.93	
Middle differentiation	30	3.58 ± 1.69	
Low differentiation	9	3.61 ± 1.56	
Invasion depth			<0.05
Mucous membrane	5	2.98 ± 1.19	
Muscular layer	22	3.45 ± 1.80	
Serous membrane from inner to outer	18	4.52 ± 0.66	
Blood-vessel invasion			<0.05
Yes	22	4.20 ± 0.98	
No	23	3.16 ± 1.01	
Lymphatic metastasis			<0.01
Yes	30	4.17 ± 1.11	
No	15	3.01 ± 0.29	
Distant metastasis			<0.01
Yes	6	5.11 ± 0.34	
No	39	3.52 ± 1.36	
TNM stage			<0.05
I, II	17	3.05 ± 0.87	
III, IV	28	4.12 ± 1.06	

^a^ Values are mean ± S.D.
